# CcWRKY31 Regulates Postharvest Mandarin Fruit Coloration in a Temperature-Dependent Manner

**DOI:** 10.3390/ijms27198798

**Published:** 2026-10-01

**Authors:** Shufang Deng, Lilei Wang, Qian Liu, Leixuan Peng, Lili Deng

**Affiliations:** 1 College of Food Science, Southwest University, Chongqing 400715, Chinalileiwangswu@gmail.com (L.W.);; 2 China-Hungary Belt and Road Joint Laboratory on Food Science, College of Food Science, Southwest University, Chongqing 400715, China

**Keywords:** WRKY transcription factor, temperature-responsive, mandarin fruit, coloration, chlorophyll, carotenoid

## Abstract

Storing mandarin fruit at 25 °C accelerates postharvest coloration of mandarin fruit, while 32 °C inhibits it. From the mandarin fruit stored at these two temperatures, we identified the temperature-responsive transcription factor CcWRKY31 as a key regulator of pigment metabolism. CcWRKY31 localizes to the nucleus and exhibits transcriptional activation activity in tobacco cells but not in yeast. Its transient overexpression in tobacco leaves caused visible yellowing and significantly reduced chlorophyll and its metabolites, with faster yellowing at 25 °C than at 32 °C. In mandarin fruit peel, transient overexpression of *CcWRKY31* induced distinct color change around the injection site and strongly upregulated *CcWRKY31* expression at both temperatures, though the induction was markedly lower at 32 °C. This overexpression promoted substantial accumulation of carotenoids and their metabolites while accelerating chlorophyll degradation. Mechanistically, CcWRKY31 directly binds to the W-box in the promoters of carotenoid biosynthesis genes (*CcCRTZ*, *CcLCYE*, *CcCCS*, *CcZEP*, *CcNCED5*) and chlorophyll degradation genes (*CcNYC1*, *CcRCCR*) to positively activate their expression. These results suggest that at the optimal temperature of 25 °C, CcWRKY31 accelerates postharvest mandarin peel coloration by coordinately promoting chlorophyll breakdown and carotenoid accumulation.

## 1. Introduction

Citrus is one of the world’s leading fruit crops in terms of cultivation area, total production, and consumer base, and it is also among the most widely cultivated and economically important fruit trees in China [1]. In China, fresh-eating citrus accounts for over 90% of total citrus production and is favored by consumers for its unique flavor and rich nutritional content [2]. Peel coloration constitutes a critical determinant for evaluating both the ripening stage and the commercial desirability of citrus fruits destined for the fresh-market segment [3]. However, problems such as regreening in summer oranges and asynchronous coloration of the peel and flesh in mandarin fruits severely reduce their commercial value and marketability [4].

Temperature is a critical environmental factor affecting citrus peel coloration by modulating pigment metabolic pathways, including chlorophyll degradation, carotenoid accumulation, and anthocyanin synthesis, thus influencing the fruit’s visual quality and commercial value [5]. Both high and low temperatures have been shown to delay color development in postharvest mandarin fruit [6], and excessively high growing temperatures result in poor coloration in *Shiranui citrus* fruit [7]. In pear [8], apple [9,10], and sweet cherry [11], low temperatures promote anthocyanin accumulation, whereas high temperatures reduce anthocyanin content.

Peel color development in citrus represents a sophisticated physiological–biochemical process, subject to transcriptional regulation by numerous transcription factors, including MYB [12,13], bHLH [14,15], WRKY, ERF [16], HSF [17], and MADS-box [18,19]. Among these, the WRKY family—one of the largest transcription factor families in plants—plays an important role in the regulatory network controlling fruit color development [20]. In citrus, *CrWRKY42* functions as a pivotal transcription factor that concurrently upregulates chlorophyll degradation and carotenoid biosynthesis. It orchestrates these two pigment pathways by directly binding to and transactivating the promoters of genes encoding chlorophyll-degrading enzymes and carotenoid synthases [21]. In sweet cherry, light-induced *PavWRKY18* directly activates key structural genes in the anthocyanin biosynthesis pathway, thereby promoting fruit coloration [11]. In wild strawberry (*Fragaria vesca*), *FvWRKY50* not only regulates vegetative growth but also controls fruit color by modulating anthocyanin accumulation; its gene-edited knockout leads to delayed anthocyanin accumulation in the fruit [22].

In this study, we identified *CcWRKY31*, a WRKY transcription factor that governs postharvest coloration in mandarin fruits, thereby extending the current functional characterization of WRKY family members in citrus fruit pigmentation.

## 2. Results

### 2.1. Effects of Different Storage Temperatures on the Expression Level of CcWRKY31 in Mandarin Fruit Peel

**Figure 1:**
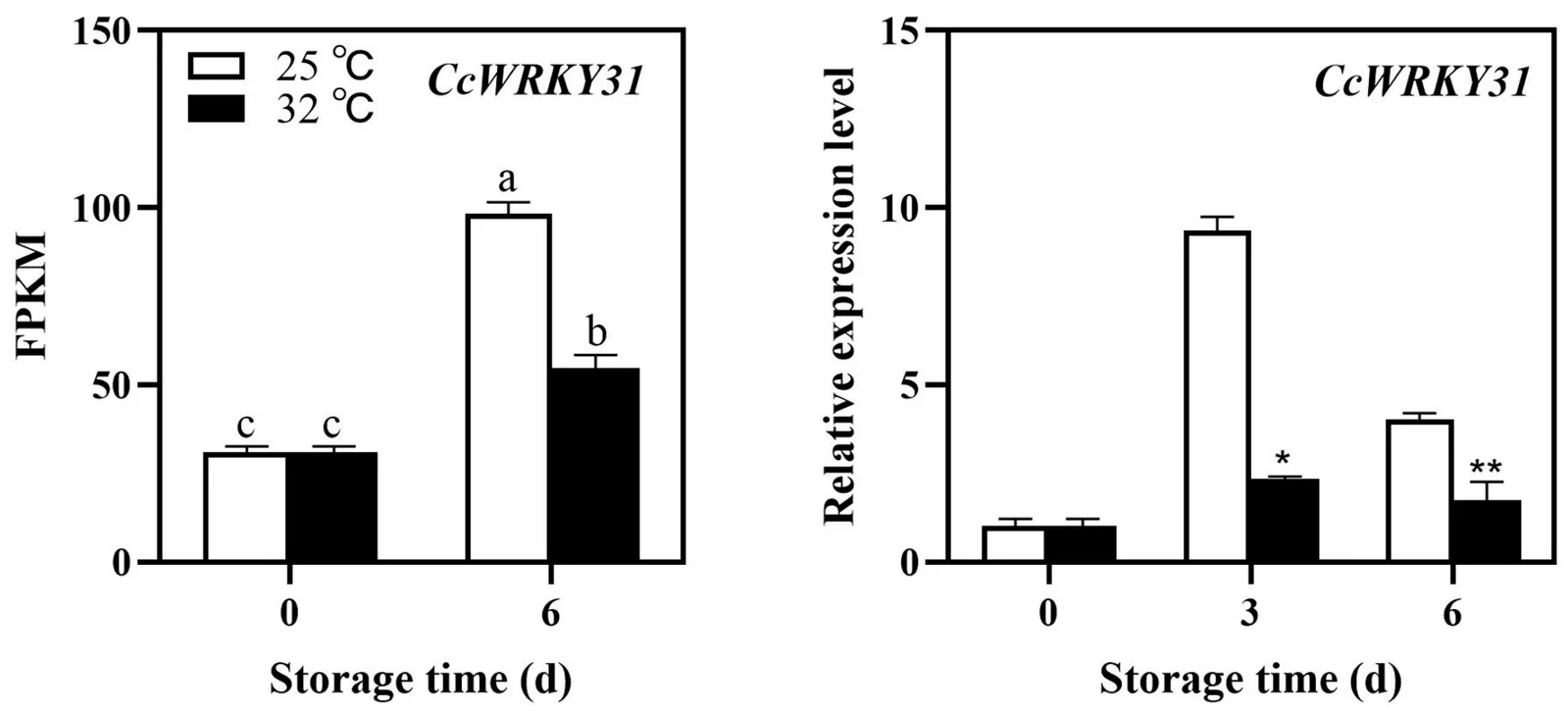
Effect of different storage temperatures on the expression of *CcWRKY31* in mandarin fruit peel. Significant differences were analyzed using Duncan’s test for the left panel and Student’s *t*-test for the right panel. In the left panel, different lowercase letters indicate significant differences at *p* < 0.05. In the right panel, * and ** indicate significant differences at *p* < 0.05 and *p* < 0.01, respectively.

Previously, our group found that storage temperature significantly affects the postharvest color development of mandarin fruit: storage at 25 °C promotes normal coloration, whereas storage at 32 °C inhibits it [6]. Transcriptomic data showed that after 6 d of storage, the expression of *CcWRKY31* (Ciclev10014642m.g, LOC18050472) was upregulated in both temperature groups. Moreover, its expression in the peel was significantly higher at 25 °C than at 32 °C (*p* < 0.05) (Figure 1), suggesting that CcWRKY31 may play a key role in the temperature-dependent differences in postharvest color development of mandarin fruit.

### 2.2. Bioinformatics Analysis of CcWRKY31

**Figure 2:**
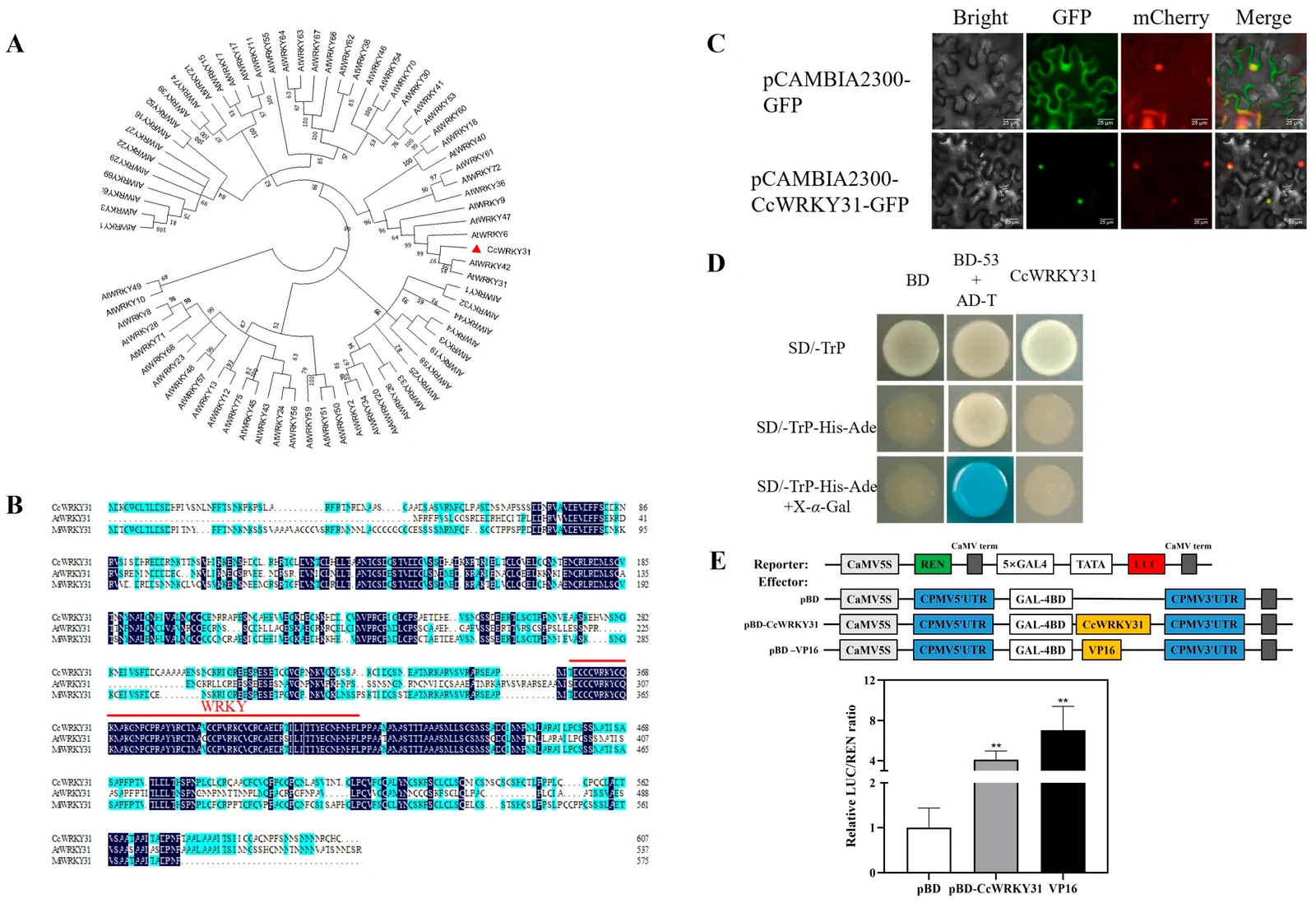
Bioinformatics analysis of *CcWRKY31.* (**A**) Phylogenetic tree of *CcWRKY31*. (**B**) Multiple sequence alignment of CcWRKY31. (**C**) Subcellular localization of *CcWRKY31* in tobacco cells. (**D**) Transcriptional activation of *CcWRKY31* in a yeast system. (**E**) Transcriptional activation of *CcWRKY31* in a tobacco system. Significant differences were analyzed using Student’s *t*-test, ** *p* < 0.01.

A phylogenetic tree was constructed by aligning the *CcWRKY31* sequence with various *Arabidopsis* amino acid sequences. The results showed that *CcWRKY31* from mandarin fruit exhibits high sequence homology with *AtWRKY31* from *Arabidopsis* (Figure 2A). Analysis of the CcWRKY31 protein sequence revealed that it consists of 608 amino acids, with a predicted molecular mass of 65.92 kDa and an isoelectric point of 6.20. A comparison of the amino acid sequences of CcWRKY31 with those of WRKY31 transcription factors from the model plant *Arabidopsis thaliana* and the extensively studied mango revealed high sequence similarity among the three proteins. All three contain the WRKY domain, belong to the WRKY family, and show a high degree of conservation (Figure 2B). Previous studies have demonstrated that AtWRKY31 in Arabidopsis participates in abscisic acid signaling during plant ripening and senescence. Given the high sequence similarity between *CcWRKY31* and *AtWRKY31*, it is plausible that CcWRKY31 may also be involved in regulating the ripening and senescence of mandarin fruit.

For subcellular localization analysis, the pCAMBIA2300-*CcWRKY31*-GFP fusion construct and the pCAMBIA2300-GFP empty vector (control) were transiently expressed in tobacco epidermal cells. Red fluorescent protein (mCherry) was used as a nuclear marker to indicate the position of the nucleus. Subcellular localization analysis revealed that the CcWRKY31-GFP fusion protein emitted green fluorescence exclusively in the nucleus, and this signal was co-incident with the mCherry marker (Figure 2C). In contrast, the GFP fluorescence of the empty vector control was distributed throughout the entire cell. These results indicate that CcWRKY31 is a nuclear-localized protein.

To examine the transcriptional activation activity of CcWRKY31, we employed a yeast transformation system together with a dual-luciferase reporter assay. As shown in Figure 2D, the positive control (BD-53+AD-T) grew well on the single-deficient medium SD/-Trp and the triple-deficient medium SD/-Trp-His-Ade, and catalyzed X-a-Gal to produce a blue color; whereas yeast cells containing BD-*CcWRKY31*, as well as the negative control (BD), failed to grow normally on SD/-Trp-His-Ade plates and could not catalyze X-a-Gal, indicating that *CcWRKY31* lacks self-activation activity in yeast. Dual-luciferase assay results (Figure 2E) show that, compared with the negative control (pBD), both *CcWRKY31* and the positive control VP16 significantly activated the expression of the LUC reporter gene, indicating that *CcWRKY31* possesses transcriptional activation activity in tobacco.

### 2.3. Effects of Transient Overexpression of CcWRKY31 on Tobacco Leaves

**Figure 3:**
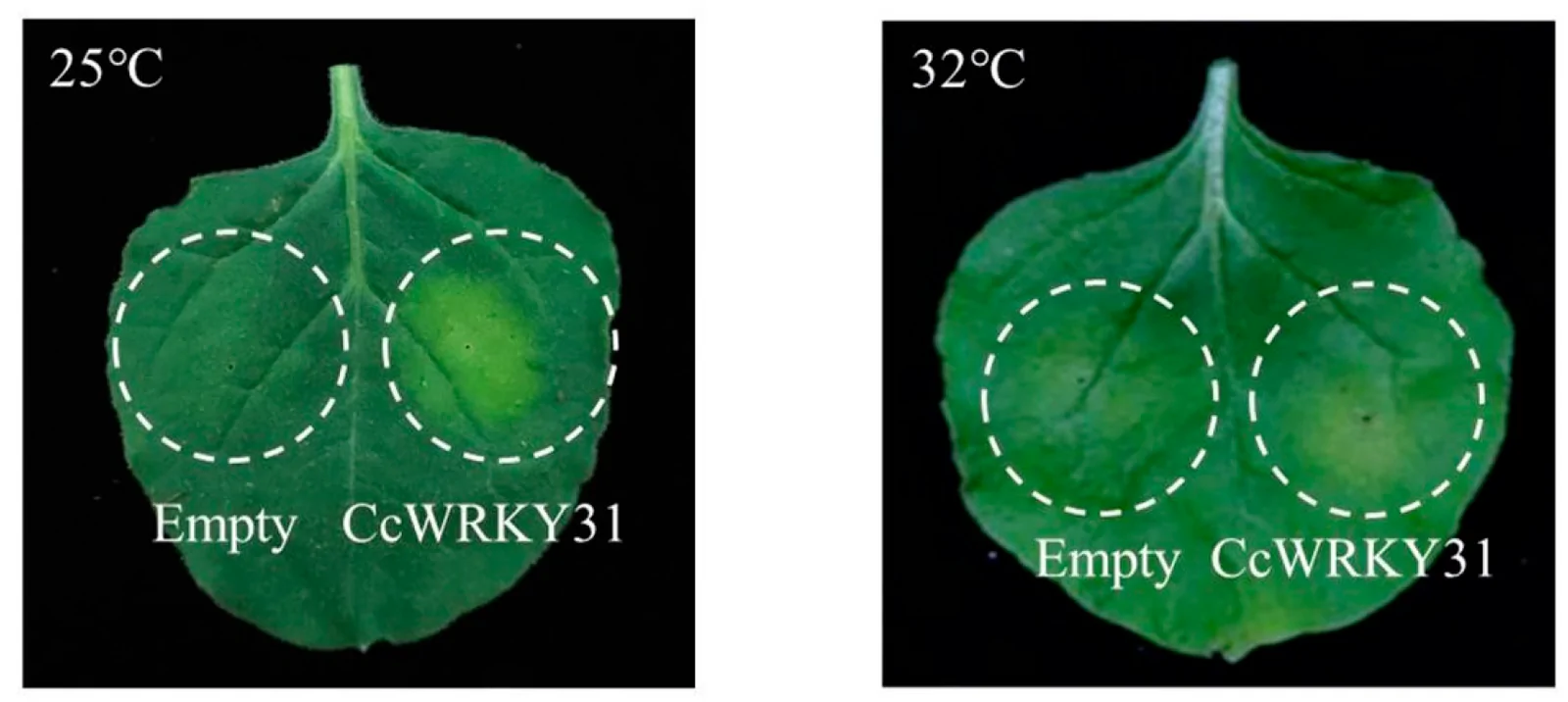
Changes in appearance of tobacco leaves after transient overexpression of *CcWRKY31* for 4 d.

The results showed that on day 4 after injection, tobacco leaves transiently overexpressing *CcWRKY31* exhibited marked yellowing at both 25 °C and 32 °C, whereas the negative control leaves remained green; yellowing progressed faster at 25 °C than at 32 °C (Figure 3).

**Figure 4:**
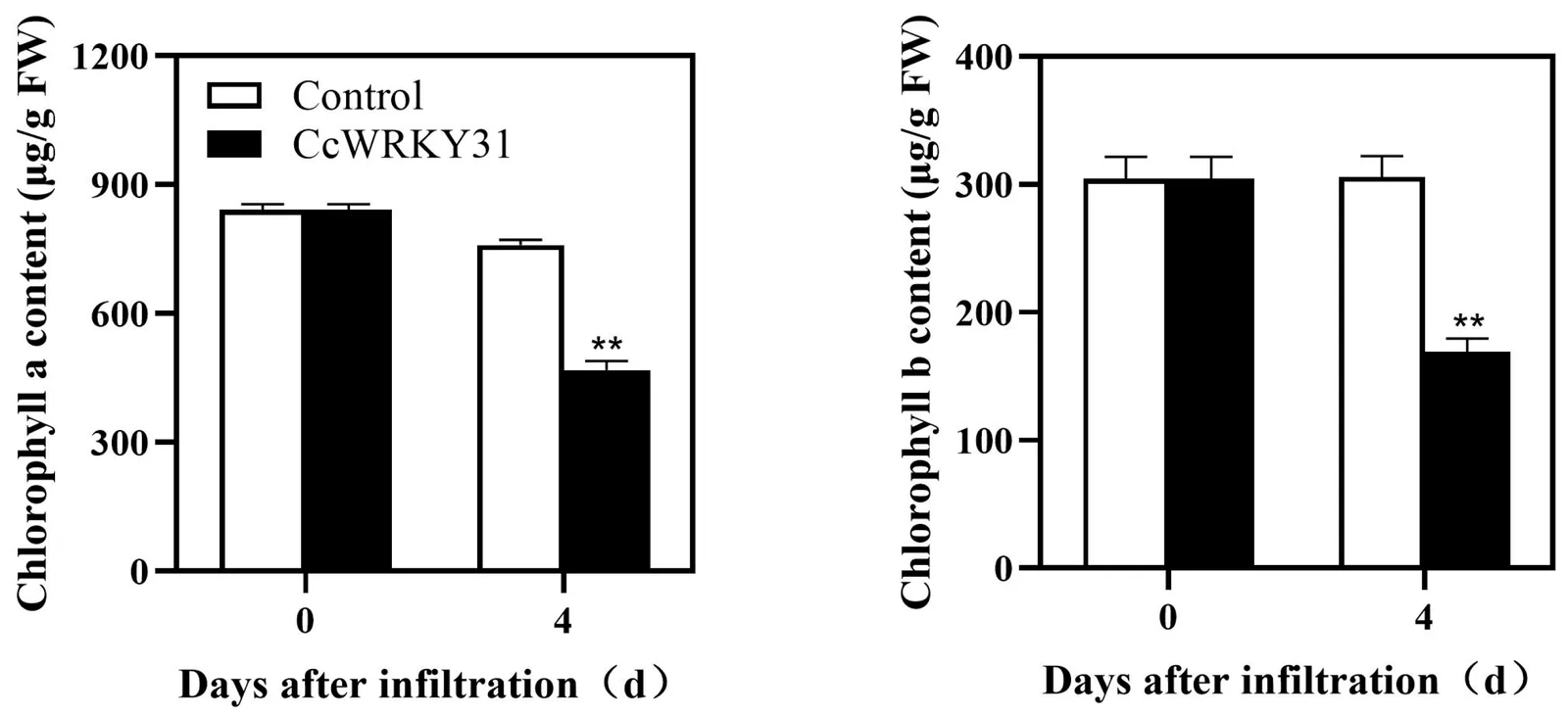
Changes in chlorophyll content of tobacco leaves after transient overexpression of *CcWRKY31* (25 °C). Significant differences were analyzed using Student’s *t*-test, ** *p* < 0.01.

As shown in Figure 4, the levels of chlorophyll a and chlorophyll b in the overexpressing leaves were reduced by 38.30% and 44.68% (*p* < 0.05), respectively, which is consistent with the observed phenotype.

### 2.4. Effects of Transient Overexpression of CcWRKY31 on Postharvest Color Development in Mandarin Fruit

#### 2.4.1. Effects of Different Storage Temperatures on *CcWRKY31* Expression Levels in Mandarin Fruit Peel

**Figure 5:**
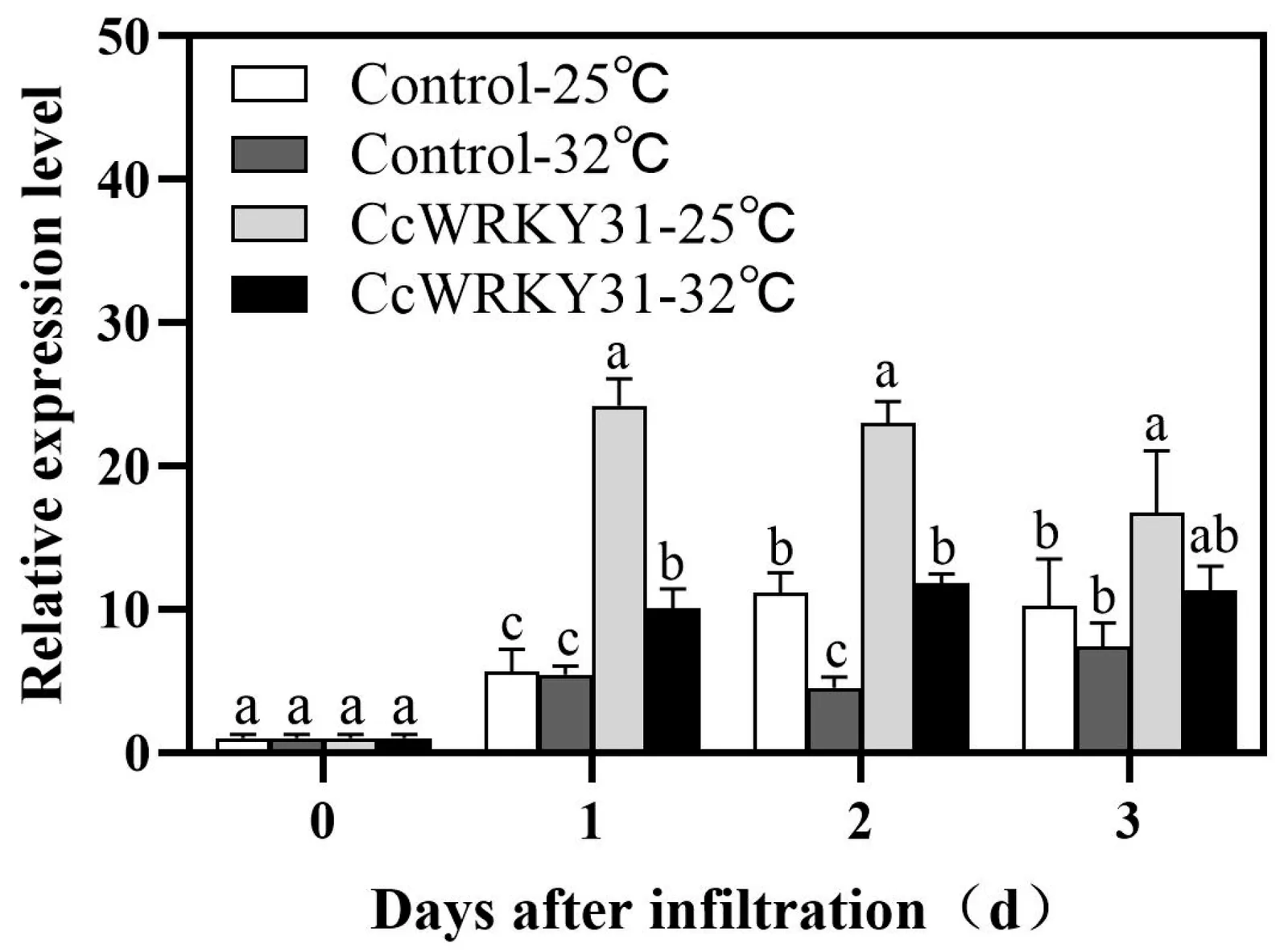
Effect of different storage temperatures on the expression of *CcWRKY31* in mandarin fruit peel after transient overexpression. Significant differences were analyzed using Duncan’s test. Different lowercase letters indicate significant differences at *p* < 0.05.

To investigate the effect of storage temperature on *CcWRKY31* expression in mandarin fruit peel, fruit transiently overexpressing *CcWRKY31* were stored at constant temperatures of 25 °C and 32 °C. Changes in *CcWRKY31* expression in the peel following transient overexpression were measured by RT-qPCR. The results showed that, compared with the negative control (Control), *CcWRKY31* expression was significantly upregulated at both 25 °C and 32 °C (*p* < 0.05) (Figure 5), confirming successful overexpression of the transcription factor at the transcript level.

During the first three days of storage (1–3 d), *CcWRKY31* expression at 32 °C was reduced by 58.62%, 48.44%, and 32.40%, respectively, relative to that at 25 °C (*p* < 0.05). These results indicate that, following transient overexpression, *CcWRKY31* expression in the peel differed markedly between the two storage temperatures, with the higher temperature (32 °C) suppressing its expression.

#### 2.4.2. Effects of Transient Overexpression of *CcWRKY31* on the Color Development of Mandarin Fruits

**Figure 6:**
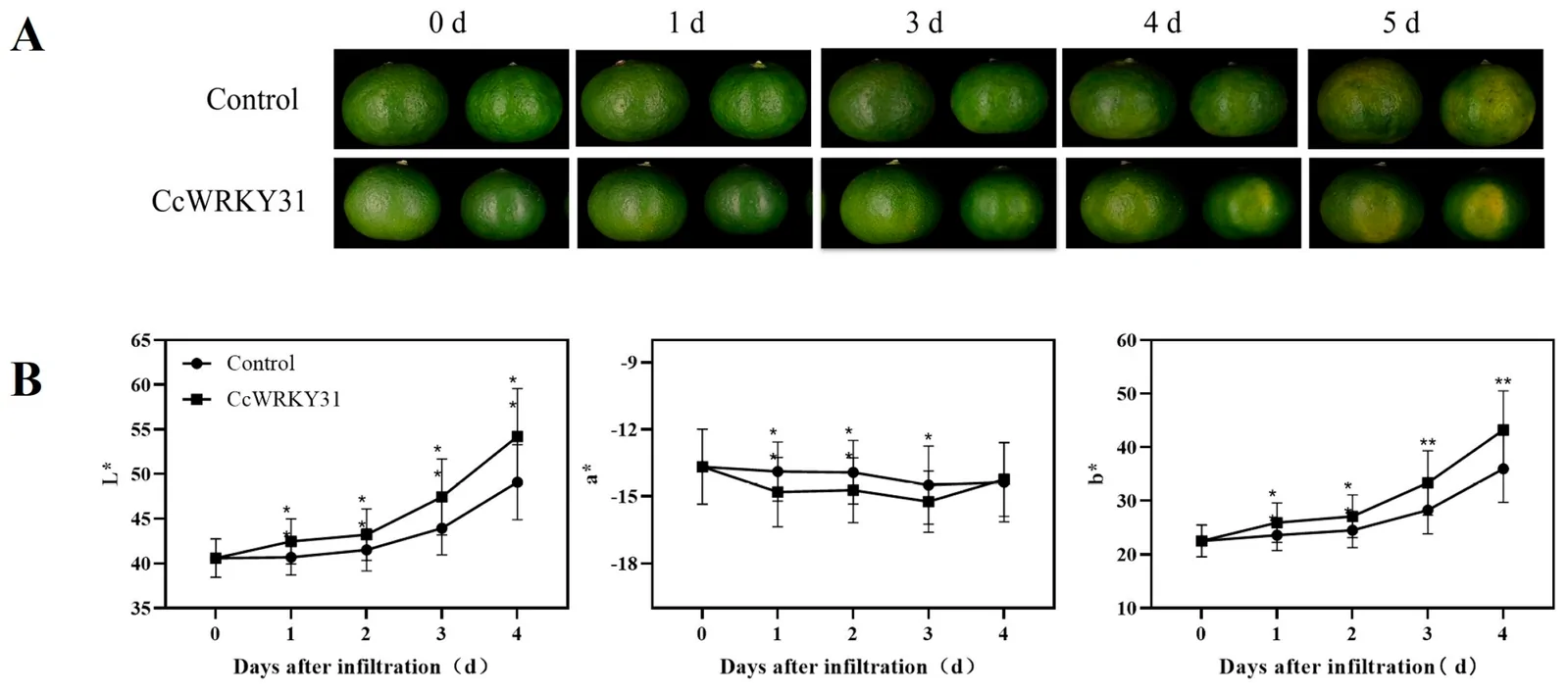
Changes in appearance (**A**) and color index (**B**) of mandarin fruit peel after transient overexpression of *CcWRKY31* (25 °C). Significant differences were analyzed using Student’s *t*-test, * *p* < 0.05, ** *p* < 0.01.

To elucidate the role of CcWRKY31 in mandarin peel pigmentation, we utilized a transient overexpression system delivered by *Agrobacterium* strain EHA105. After injection with an *Agrobacterium* EHA105 suspension and incubation at 25 °C for 3 d, the mandarin fruit peel transgenically expressing *CcWRKY31* exhibited a noticeable color change near the injection site, whereas the peel of the negative control remained green (Figure 6A). Accordingly, the L* and b* values of the mandarin fruit peel transgenically overexpressing *CcWRKY31* were significantly higher (*p* < 0.01) than those of the negative control; on day 4, the L*, a*, and b* values in the *CcWRKY31* group increased by 9.91%, 5.24%, and 18.26%, respectively, compared with the control group (Figure 6B).

#### 2.4.3. Effects of *CcWRKY31* Transient Overexpression on Carotenoid and Metabolite Levels in Mandarin Fruit Peel

**Figure 7:**
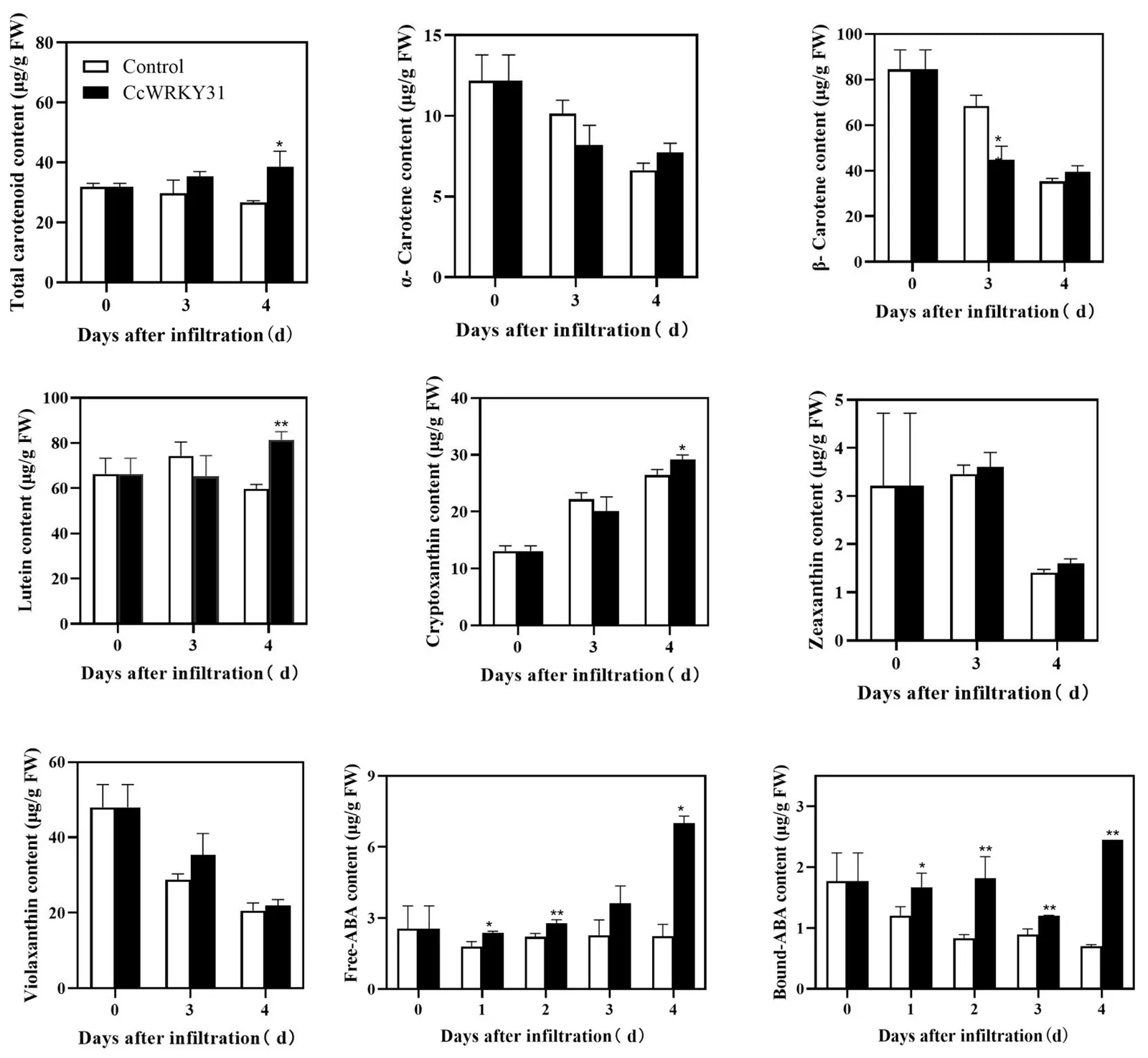
Effects of transient overexpression of *CcWRKY31* on the content of carotenoid and its metabolites in the peel of mandarin fruit. Significant differences were analyzed using Student’s *t*-test, * *p* < 0.05, ** *p* < 0.01.

*CcWRKY31* transient overexpression triggered a sustained rise in total carotenoid, lutein, and cryptoxanthin concentrations over the storage period. After 4 days, the accumulation of these compounds in the transformed peel surpassed that of the control by 45.15%, 36.58%, and 10.36%, respectively (Figure 7). As shown in Figure 7, following transient overexpression of *CcWRKY31*, the free ABA content in the mandarin fruit peel continued to rise as storage time increased. By day 4, it was significantly higher than that of the control group, reaching 2.14 times the control level (*p* < 0.01). In contrast, the bound abscisic acid content in the control group showed a continuous downward trend, while the free abscisic acid content remained unchanged (Figure 7).

#### 2.4.4. Effects of Transient Overexpression of *CcWRKY31* on Chlorophyll in Mandarin Fruit Peel

**Figure 8:**
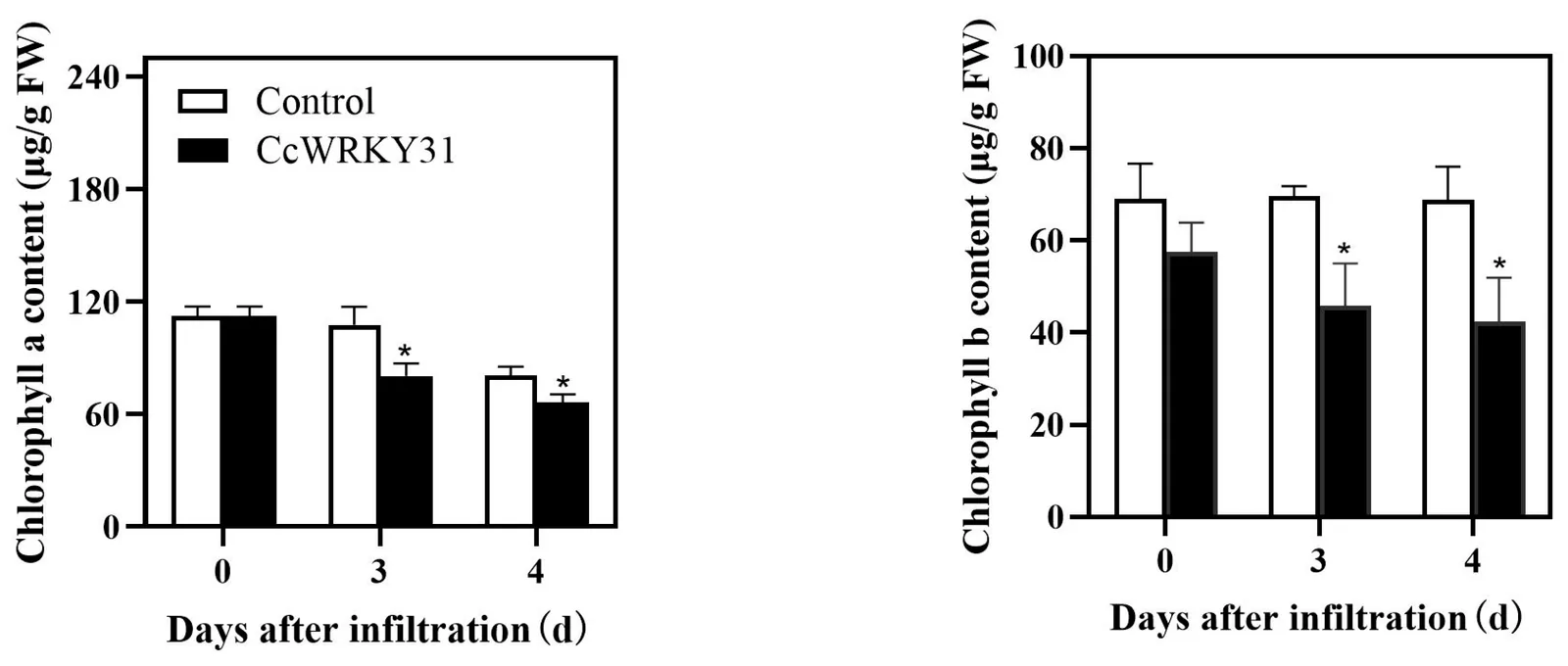
Effects of transient overexpression of *CcWRKY31* on the content of chlorophyll and its metabolites in the peel of mandarin fruit. Significant differences were analyzed using Student’s *t*-test, * *p* < 0.05.

The results showed that, as storage time increased, the levels of chlorophyll and its degradation products in the peel of mandarin fruits overexpressing *CcWRKY31* continued to decrease compared with the negative control (*p* < 0.05) (Figure 8). On day 4, the levels of chlorophyll a and chlorophyll b were 17.80% and 38.49% lower, respectively, than those in the control group (*p* < 0.05).

### 2.5. Screening of Potential Interacting Target Genes of CcWRKY31

#### 2.5.1. Identification of Potential Target Genes of CcWRKY31 by Transcriptomic Analysis

**Figure 9:**
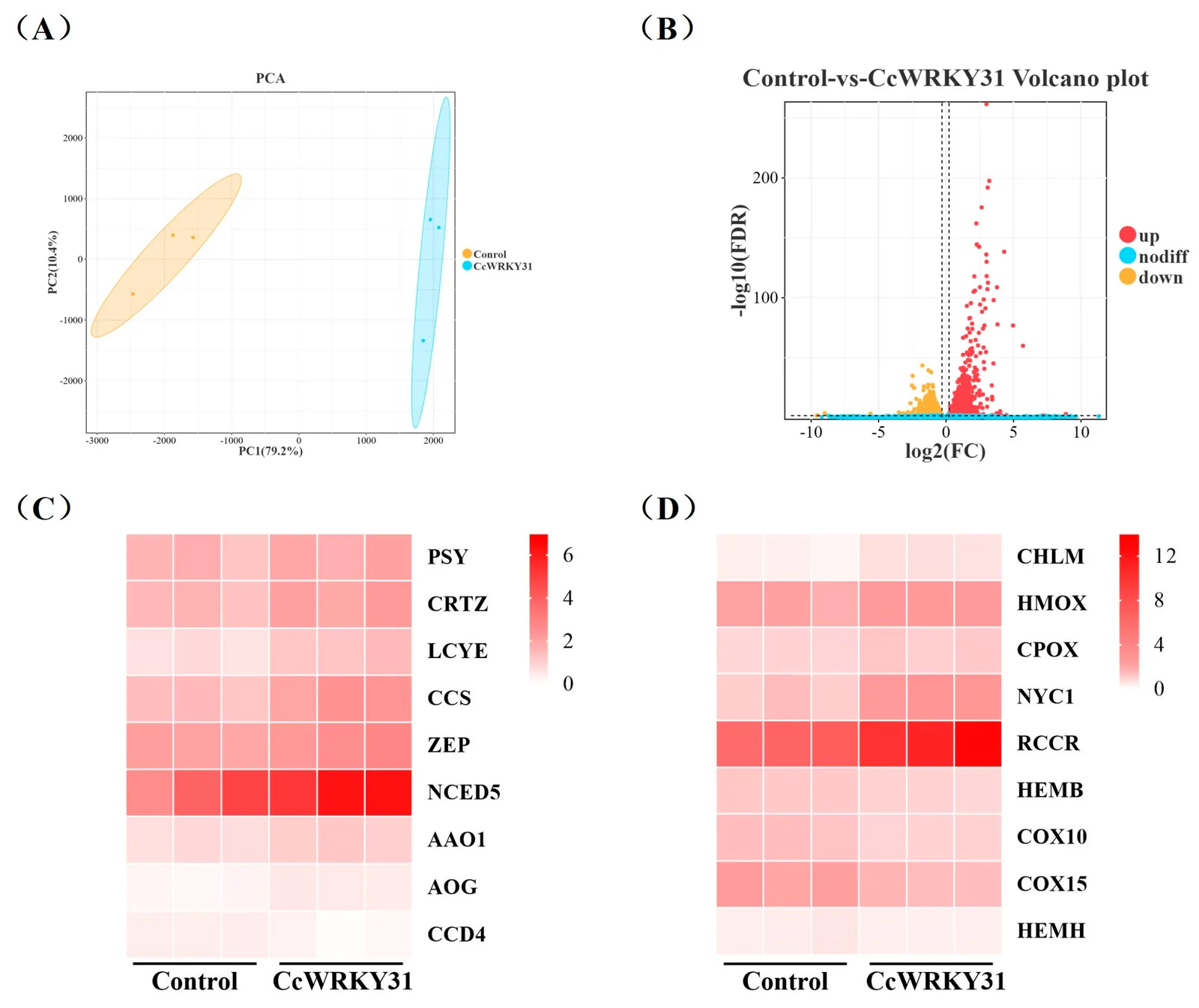
RNA seq analysis of transient overexpression of *CcWRKY31* in mandarin fruit peel. (**A**) The PCA analysis of component of control and *CcWRKY31* samples. (**B**) The distribution of DEGs between control and *CcWRKY31* groups. (**C**) Fold change of differentially expressed genes in the carotenoid biosynthesis pathway from RNA seq. (**D**) Fold change of differentially expressed genes in the chlorophyll degradation pathway from RNA seq.

To further elucidate the expression of genes regulated by *CcWRKY31*, transcriptomic sequencing (RNA-seq) analysis was performed on mandarin fruit peel that had been transiently overexpressing *CcWRKY31* for 2 d. Differentially expressed genes (DEGs) were analyzed using an FDR < 0.05 as the screening criterion. Compared with the control group, a total of 3702 differentially expressed genes were identified, of which 1909 were upregulated and 1793 were downregulated (Figure 9A,B). After 2 d of transient overexpression, both carotenoid and chlorophyll metabolism in the mandarin fruit peel were affected; specifically, nine DEGs were enriched in the carotenoid metabolic pathway, and nine DEGs were enriched in the chlorophyll metabolic pathway (Figure 9C,D). See the Supplementary figures for the GO enrichment analysis (Figure S1) and the KEGG enrichment analysis (Figure S2).

#### 2.5.2. Effects of Transient Overexpression of *CcWRKY31* on the Expression of Genes Related to Pigment Metabolism

**Figure 10:**
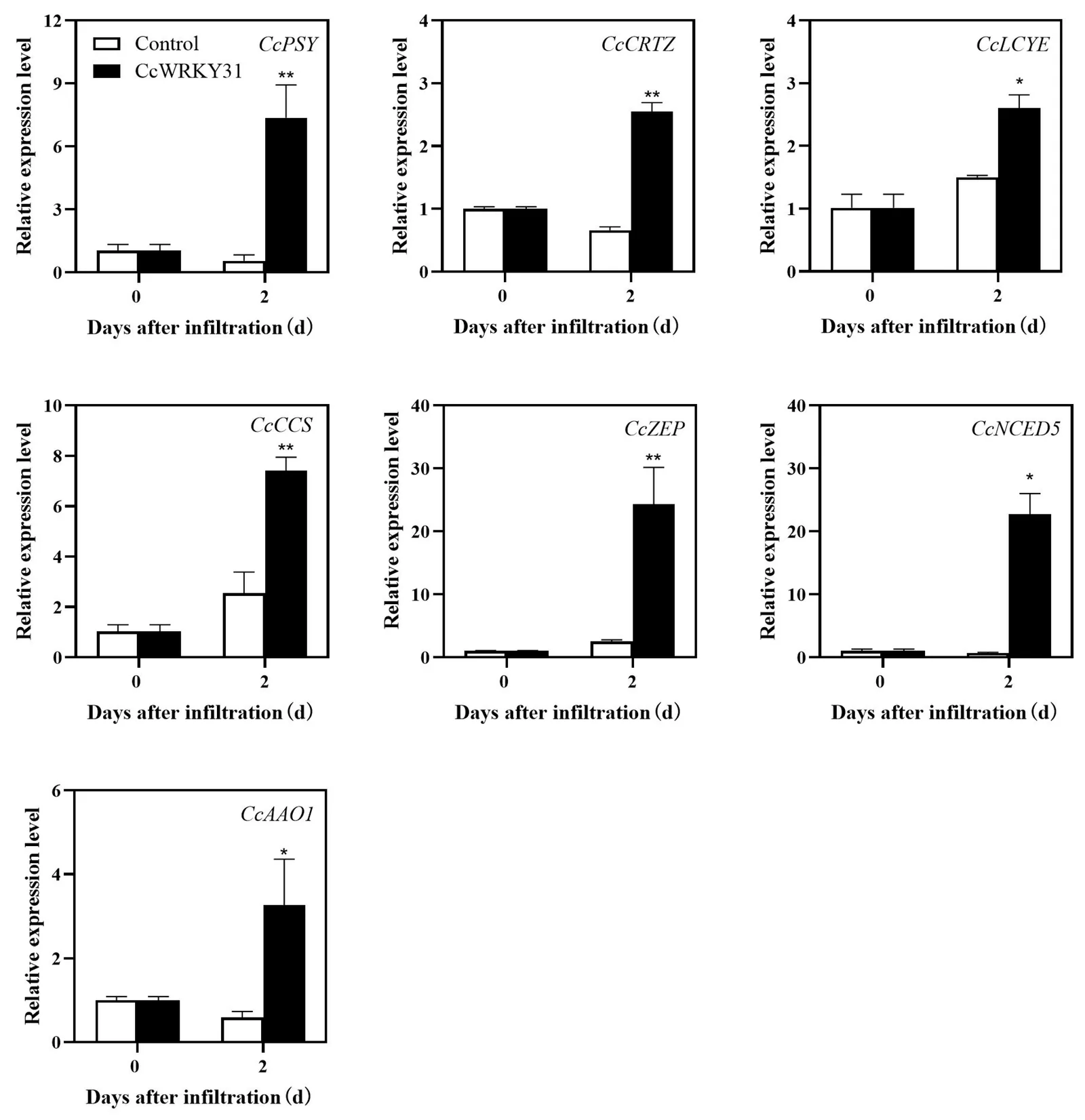
The effect of transient overexpression of *CcWRKY31* on the expression of carotenoid metabolism related genes. Significant differences were analyzed using Student’s *t*-test, * *p* < 0.05, ** *p* < 0.01.

**Figure 11:**
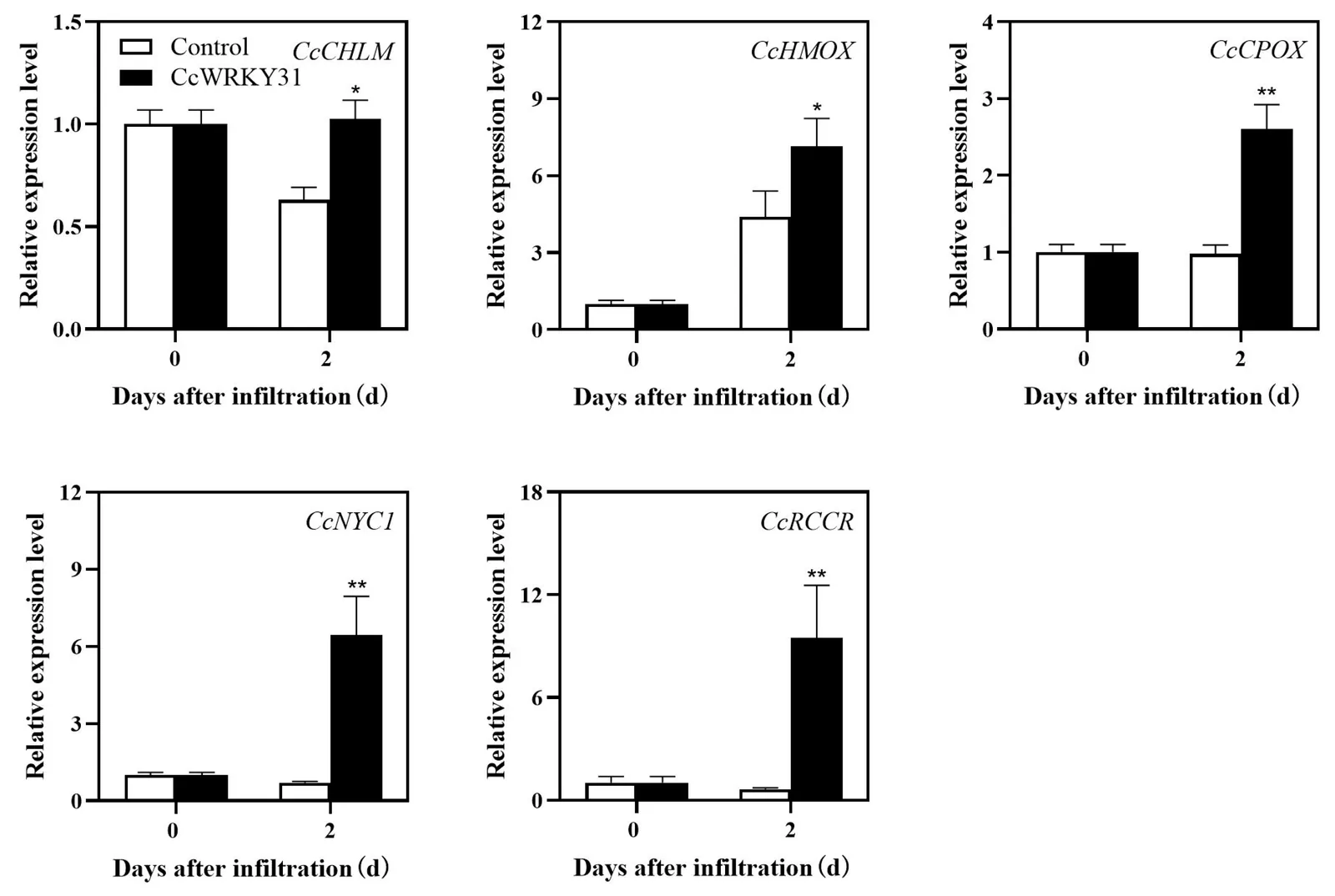
The effect of transient overexpression of *CcWRKY31* on the expression of chlorophyll metabolism related genes. Significant differences were analyzed using Student’s *t*-test, * *p* < 0.05, ** *p* < 0.01.

RT-qPCR results showed that seven genes involved in the carotenoid biosynthesis pathway (*CcPSY*, *CcCRTZ*, *CcLCYE*, *CcCCS*, *CcZEP*, *CcNCED5*, *CcAAO1*) (Figure 10) and five genes involved in the chlorophyll metabolism pathway (*CcCHLM*, *CcHMOX*, *CcCPOX*, *CcNYC1*, *CcRCCR*) were significantly upregulated (*p* < 0.05) in the treated fruit groups (Figure 11). These results suggest that *CcWRKY31* may positively regulate carotenoid accumulation and chlorophyll degradation by increasing the expression of genes involved in carotenoid biosynthesis and chlorophyll degradation in the peel of mandarin fruits, thereby promoting fruit coloration.

#### 2.5.3. CcWRKY31 Directly Binds to and Activates the Promoters of Key Genes Involved in Carotenoid Biosynthesis

**Figure 12:**
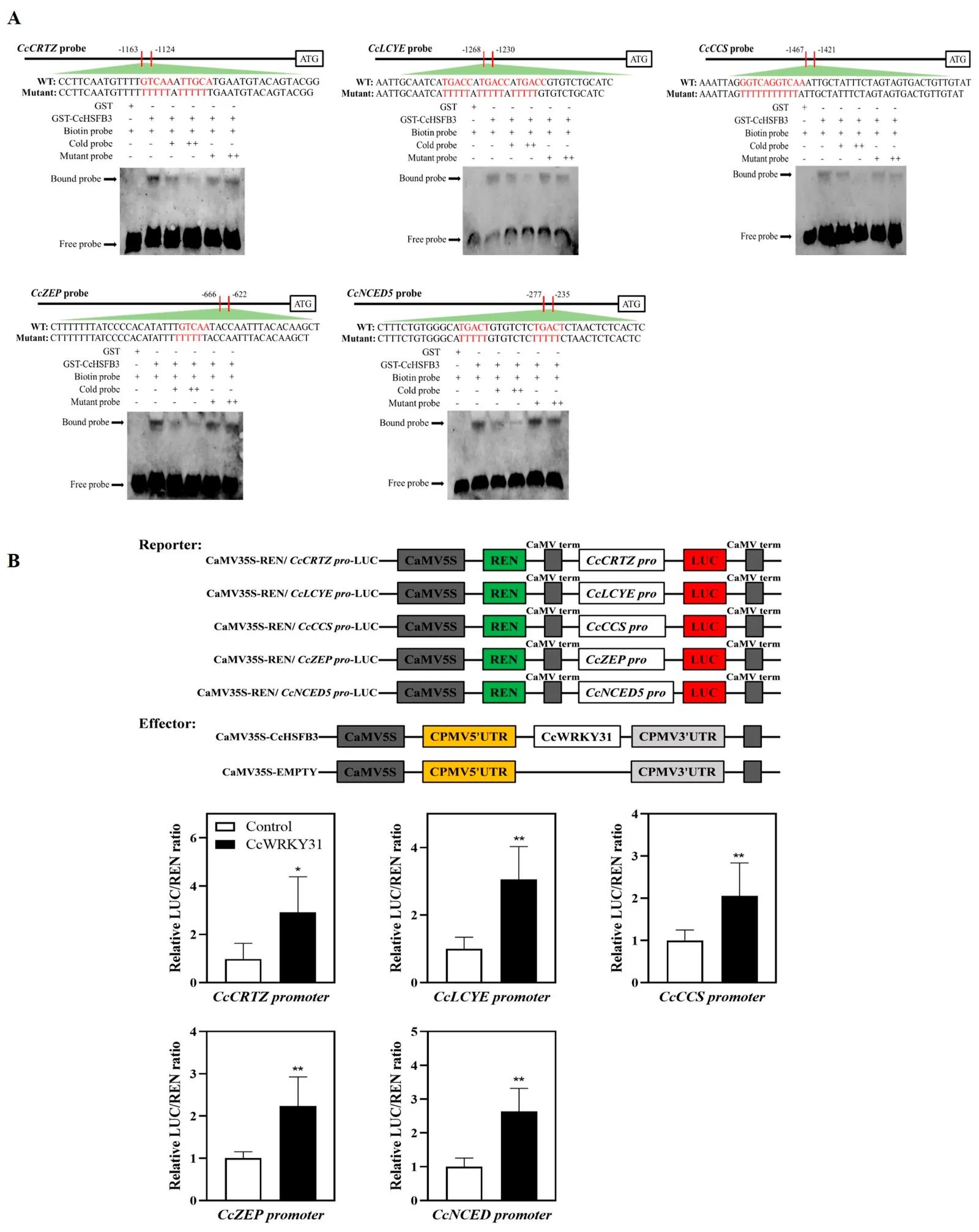
The regulatory effect of *CcWRKY31* on key genes involved in carotenoid biosynthesis. (**A**) EMSA analysis of the bindings of GST-*CcWRKY31* to the W-box motif in promoters of *CcCRTZ*, *CcLCYE*, *CcCCS*, *CcZEP* and *CcNCED5*. (**B**) Dual-luciferase transient expression assay of *CcWRKY31* on target genes in tobacco leaves. Significant differences were analyzed using Student’s *t*-test, * *p* < 0.05, ** *p* < 0.01.

Analysis of the promoter regions of *CcCRTZ*, *CcLCYE*, *CcCCS*, *CcZEP*, and *CcNCED5* revealed that they all contain varying numbers of W-box elements, as verified by electrophoretic mobility shift assay (EMSA) and dual-luciferase reporter assay (DLR). As shown in Figure 12A, the *CcWRKY31* recombinant protein directly binds to the W-box elements in the promoter regions of *CcCRTZ*, *CcLCYE*, *CcCCS*, *CcZEP*, and *CcNCED5*, resulting in a binding band that co-migrates with the labeled DNA probe. At the same time, as the concentration of unlabeled competitive DNA probes (cold probes) with the same sequence increased, the migration bands disappeared; however, the concentration of the mutant probe (AAAA/TTTT) used did not affect the binding bands. In addition, a dual-luciferase reporter system was constructed to analyze the transcriptional activation of these genes by *CcWRKY31*. (Figure 12B) shows that, compared with the control group (pEAQ and Reporter co-transformed into tobacco), the LUC/REN ratios were significantly higher (*p* < 0.05) when pEAQ-*CcWRKY31* was co-transformed into tobacco with *CcCRTZ*, *CcLCYE*, *CcCCS*, *CcZEP*, and *CcNCED5*. These results indicate that *CcWRKY31* can specifically bind to the W-box in the promoters of these genes and activate their expression.

#### 2.5.4. CcWRKY31 Directly Binds to and Activates the Promoters of Key Genes Involved in Chlorophyll Degradation

**Figure 13:**
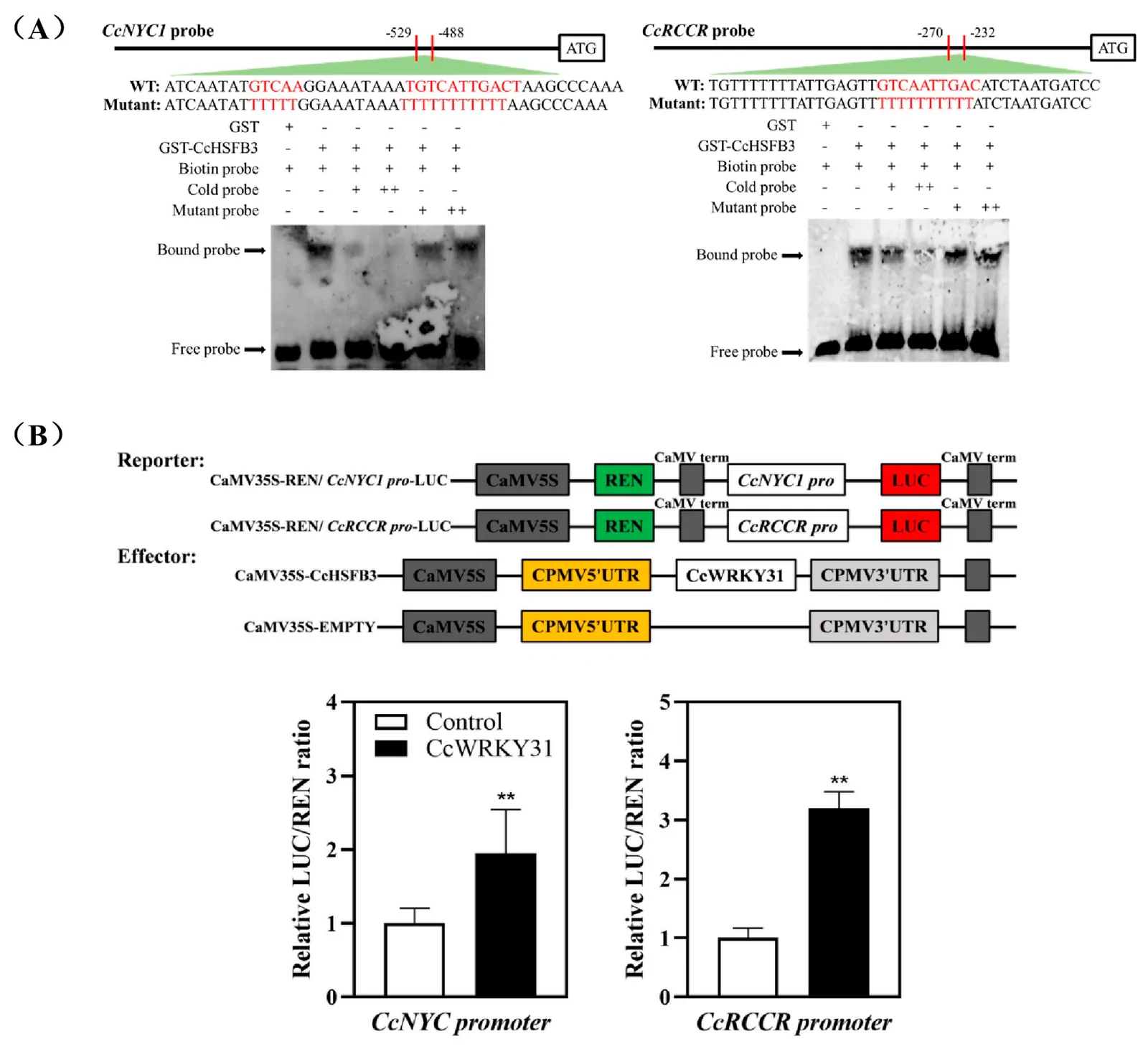
The regulatory effect of *CcWRKY31* on key genes involved in chlorophyll degradation. (**A**) EMSA analysis of the bindings of GST-*CcWRKY31* to the W-box motif in promoters of *CcNYC*, *CcRCCR*. (**B**) Dual-luciferase transient expression assay of *CcWRKY31* on target genes in tobacco leaves. Significant differences were analyzed using Student’s *t*-test, ** *p* < 0.01.

Bioinformatics analysis revealed that the promoters of both *CcNYC1* and *CcRCCR* contain varying numbers of W-box elements. EMSA and DLR assays demonstrated that the *CcWRKY31* recombinant protein binds to promoter fragments of these two genes, resulting in changes in migration rates (Figure 13A). As the amount of unlabeled competitive DNA probe (cold probe) with the same sequence increased, the binding ability of the recombinant *CcWRKY31* protein to the biotin-labeled probe decreased significantly. In contrast, the binding between the two was not affected by the mutant probe (AAAA/TTTT). Dual-luciferase reporter assay results showed that, for the reporter genes constructed from *CcNYC1* and *CcRCCR*, *CcWRKY31* significantly increased the LUC:REN ratio compared with the control (pEAQ) (*p* < 0.05) (Figure 13B). These results indicate that *CcWRKY31* can specifically bind to the W-box in the *CcNYC1* and *CcRCCR* promoters and activate their expression.

## 3. Discussion

WRKY transcription factors constitute one of the largest families of transcriptional regulators involved in diverse developmental processes in plants, animals, and fungi [23], with particularly prominent roles in flower and fruit development [24]. Members of this family have been shown to directly or indirectly modulate the expression of genes associated with ripening, senescence, and pigment metabolism in species such as osmanthus [25], tomato [26], and sorghum [27], thereby influencing chlorophyll degradation, carotenoid biosynthesis, and fruit ripening. Our findings broaden the functional repertoire of this family: we identified *CcWRKY31* as a nuclear-localized transcriptional activator with high similarity to *Arabidopsis AtWRKY31*, whose transient overexpression causes chlorosis in tobacco leaves and markedly accelerates color development in mandarin fruit peel, suggesting a potential role in postharvest temperature-induced fruit coloration.

Through EMSA and dual-luciferase reporter assays, we demonstrated that CcWRKY31 directly binds to and activates the promoters of the carotenoid biosynthetic genes *CcCRTZ*, *CcLCYE*, *CcCCS*, and *CcZEP*, the chlorophyll degradation genes *CcRCCR* and *CcNYC1*, and the key abscisic acid (ABA) biosynthetic rate-limiting enzyme gene *CcNCED5*, upregulating their expression. This multi-target direct regulatory mode provides a molecular explanation for how *CcWRKY31* coordinates carotenoid accumulation and chlorophyll degradation. Compared with other transcription factors that simultaneously modulate these two pigment pathways, such as kiwifruit *AdMYB7* [28], tomato *SlBBX20* [29], and litchi *LcNAC002* [30], *CcWRKY31* displays a notably broad target spectrum: it not only directly activates both pigment anabolic and catabolic genes but also directly controls the rate-limiting step of ABA biosynthesis. In particular, the citrus *CrWRKY42* transcription factor primarily activates *CrPDS*, *CrLCYb2*, *CrNYC*, and *CrSGR* [21], whereas *CcWRKY31* additionally targets genes such as *CcCCS* and *CcNCED5*, which are not directly activated by *CrWRKY42*. This divergence in target gene profiles suggests functional specialization among citrus WRKY proteins in peel color regulation, possibly achieved through distinct *cis*-element recognition or the formation of specific transcriptional complexes with different cofactors, allowing fine-tuned responses to developmental and environmental cues.

A particularly noteworthy finding is that *CcWRKY31* directly upregulates *CcNCED5*. NCED catalyzes the cleavage of 9-*cis*-epoxycarotenoids, a rate-limiting step in ABA biosynthesis [31]. Therefore, the substantial increase in ABA content observed in transiently overexpressing peels should not be solely attributed to a substrate mass effect resulting from an enlarged carotenoid pool [32,33]; it is at least partly driven by direct transcriptional activation of *CcNCED5* by *CcWRKY31*. This establishes a tighter molecular coupling: on the one hand, *CcWRKY31* promotes the synthesis of carotenoids, including ABA precursors, and on the other hand, it actively opens the ABA biosynthetic valve. Considering that ABA is a key ripening- and senescence-promoting hormone, and that ABA signaling can in turn feedback to enhance carotenoid accumulation, we propose the existence of a positive feedback regulatory loop—ABA elevation driven by *CcWRKY31* might further sustain or amplify its own expression or activity via ABA-responsive elements, thereby accelerating chlorophyll degradation and carotenoid accumulation and propelling the fruit into an irreversible ripening and coloration program. The precise molecular details of this feedback loop, and the potential involvement of other hormones such as ethylene, await clarification through experiments including suppression of ABA signaling and dissection of hormone-responsive elements in the *CcWRKY31* promoter.

The speculation that *CcWRKY31* participates in postharvest temperature-induced coloration also opens promising avenues for future investigation. It is well documented that low temperature can induce the expression of many fruit coloration-related genes. Whether *CcWRKY31* expression is directly induced by temperature, and whether its protein stability or transcriptional activity is modulated by temperature-associated signals (e.g., calcium signaling, reactive oxygen species), are questions that can be addressed by analyzing the cis-acting elements in the *CcWRKY31* promoter, examining expression kinetics under different postharvest temperature regimes, and characterizing its protein interactome.

While this study establishes a preliminary regulatory framework for CcWRKY31, several limitations must be acknowledged. The current conclusions are largely based on transient overexpression and in vitro binding assays; the supraphysiological accumulation of the protein in transient systems may cause non-physiologically strong activation or even disrupt endogenous feedback homeostasis. Therefore, genetic approaches such as stable transformation, genome-edited knockout, or RNAi-mediated silencing are necessary to definitively validate the endogenous function of *CcWRKY31* in whole fruits or stable materials. Furthermore, although EMSA and dual-luciferase assays demonstrate direct binding and activation capacity, they cannot resolve whether this binding occurs effectively in the native chromatin context; ChIP-qPCR would be a powerful complementary approach. It will also be important to explore potential synergistic, antagonistic, or hierarchical relationships between CcWRKY31 and other known pigment regulators, such as CrWRKY42, MYB, and NAC factors.

In summary, our study reveals that *CcWRKY31* functions as a key transcriptional node that synchronously drives carotenoid biosynthesis, ABA production, and chlorophyll degradation by directly upregulating *CcCRTZ*, *CcLCYE*, *CcCCS*, *CcZEP*, *CcNCED5*, *CcNYC1*, and *CcRCCR*, potentially reinforcing the entire coloration process through an ABA-mediated positive feedback loop. This multi-layered regulatory network provides new insights into the fine coordination of pigment metabolism during fruit ripening and offers a potential molecular target for improving postharvest fruit color quality.

## 4. Materials and Methods

### 4.1. Test Materials

Fruits of the mandarin cultivar Citrus (*unshiu* Marc). at the green-ripe stage were obtained from a commercial orchard located at Jigong Mountain, Beibei District, Chongqing, during September 2020. (170 d after peak flowering) and transported to the laboratory immediately after harvest. Healthy fruits of uniform size and color, free from mechanical damage, diseases, and pests, were selected. After washing with clean water, they were air-dried at room temperature, divided into two groups of 100 fruits each, individually packaged, and stored in the dark in constant-temperature incubators at 25 and 32 °C, respectively. Each treatment consisted of 3 independent biological replicates at each time point, with each biological replicate comprising 10 fruits (at 0 d, the two treatment groups shared 3 independent biological replicates). At 0, 3, 6, and 9 d of storage, a strip of peel 1–2 cm wide was collected from the equatorial region of each fruit, rapidly minced, frozen in liquid nitrogen, and stored at −80 °C for transcriptomic sequencing.

Green-ripe fruits collected from the same orchard during September 2022 and August 2023 were rapidly brought to the laboratory, where they were washed with water, air-dried at room temperature, and subsequently utilized as explants for *Agrobacterium*-mediated transient transformation assays.

*Nicotiana benthamiana* was raised from seeds that were sown on a soil mixture of potting soil and vermiculite (3:1) and kept at 25 °C under a daily regime of 16 h light and 8 h dark, all within our laboratory.

### 4.2. Transcriptome Data Analysis

Preliminary experimental results showed that the peel of mandarin fruits turned yellow after 6 d of storage at 25 °C, whereas those stored at 32 °C remained green [6]. Therefore, peel samples collected at 0 and 6 d of storage were submitted to Guangzhou Kidio Biotechnology Co., Ltd. (Guangzhou, China) for transcriptome sequencing; library construction and transcriptome analysis were performed according to the company’s protocols. The reference genome was *Citrus_clementina*_v1.0 (GCF_000493195.1). Fold change (FC) and false discovery rate (FDR) were used as metrics for identifying differentially expressed genes, with |log_2_(FC)| > 1 and FDR < 0.05 as the selection criteria.

### 4.3. Gene Cloning

Following the method described by Wang [34], the CDS sequence of *CcWRKY31* was cloned and a recombinant vector was constructed. See Table S1 for the primers.

### 4.4. Bioinformatics Analysis

#### 4.4.1. Amino Acid Sequence Analysis and Phylogenetic Tree

Following the method described by Wang [34], we performed an amino acid sequence analysis of CcWRKY31 and constructed a phylogenetic tree.

#### 4.4.2. Subcellular Localization

Following the method described by Wang [34], the CDS of *CcWRKY31* was cloned into the pCAMBIA2300-GFP vector and injected into tobacco plants via transient transformation; the results were observed using an Olympus BX63 fluorescence microscope (EVIDENT, Tokyo, Japan).

#### 4.4.3. Analysis of Transcription Activation Activity in Yeast

The Y2HGold-GAL4 system was used to evaluate the transcriptional self-activation of CcWRKY31. The full-length CDS was PCR-amplified, cloned into pGBKT7, and transformed into Y2HGold competent cells (primers in Table S1). pGBKT7-53 + pGADT7-T and empty pGBKT7 served as positive (BD-53+AD-T) and negative (BD) controls, respectively. Transformants were selected on SD/-Trp medium, then replica-plated onto SD/-Trp/-His/-Ade medium containing X-α-Gal (2 g·L^−1^). Self-activation was assessed by colony growth and blue color development. Positive controls grew on all media with blue coloration, whereas negative controls grew only on SD/-Trp, confirming that CcWRKY31 possesses transcriptional activation activity.

### 4.5. Analysis of Transcription Activation Activity in Tobacco

Specific primers were designed to amplify the CDS sequence of the *CcWRKY31* transcription factor (excluding the stop codon) by PCR. The amplified fragment was cloned into the linearized pBD vector to generate the effector construct. The empty pBD vector and pBD-VP16 were used as the negative and positive controls, respectively. The dual-luciferase reporter vector pGreenII 0800-LUC, containing GAL4-LUC and Renilla luciferase (REN), served as the reporter. The effector and reporter were separately transformed into *Agrobacterium* strain EHA105. Following established methods, the effector and reporter strains were co-transformed into tobacco leaf cells at a 9:1 ratio. At 2–3 d after co-transformation, the LUC/REN ratio was measured using a dual-luciferase assay kit (Yeason, Shanghai, China). At least six independent biological replicates were performed.

### 4.6. Construction of the Overexpression Vector

The overexpression vector pCAMBIA2300-CcWRKY31 was constructed based on the method described by Wang [34]. See Table S1 for the primers.

### 4.7. Transient Overexpression in Tobacco Leaves and Mandarin Fruit Peels

Transient overexpression in tobacco leaves: pCAMBIA2300 was used as the transient overexpression vector. *Agrobacterium* culture and resuspension were performed according to the subcellular localization analysis protocol, with the mixed bacterial suspension having an OD_600_ of 0.1. Agrobacteria containing pCAMBIA2300-*CcWRKY31* and pCAMBIA2300 (empty vector control) were injected separately on either side of the midrib on the underside of Benjamina tobacco leaves.

For transient overexpression in mandarin fruits, the *Agrobacterium* culture harboring the target construct was mixed with the P19 helper culture to a final OD_600_ of 0.4. Two holes, each 1 mm in diameter and 1 mm in depth, were punctured at equidistant positions along the equatorial plane of each fruit. Each hole was injected with a 0.4 mL aliquot of the mixed bacterial suspension via a needle-free syringe. After the suspension had been fully absorbed, the fruits were individually enclosed in 170 mm × 140 mm polyethylene fruit bags (0.02 mm thick). Fruits harvested in September 2022 were maintained in the dark at 25 °C post-injection; fruits harvested in August 2023 were kept in the dark at either 25 °C or 32 °C. At 0, 1, 2, 3, and 4 d after injection, a 1 cm wide strip of peel surrounding the injection site was excised, cut into small pieces, flash-frozen in liquid nitrogen, and stored at −80 °C for subsequent analyses. Each treatment at each time point comprised three independent biological replicates, with each replicate consisting of ten fruits. Notably, at the 0d time point, the two temperature treatments shared the same method.

### 4.8. Determination of Pigment Content and Related Gene Expression in Mandarin Fruits and Tobacco

#### 4.8.1. Determination of Carotenoid and Metabolite Content in Mandarin Fruit Peel

Measurement of carotenoid and carotenoid metabolite content using Ge’s method [6].

#### 4.8.2. Determination of Chlorophyll Content in Tobacco Leaves and Mandarin Fruit Peel

Determination of chlorophyll content using the method described by Yuan and Das [35,36].

### 4.9. RT-qPCR Gene Expression Analysis

RT-qPCR experiments were performed following the method described by Wang [34]; see Table S1 for the primers.

### 4.10. EMSA Experiment

The EMSA experiment was adapted from the method described by Wang [34].

### 4.11. DLR Experiment

We cloned the target gene promoter into the pGreenII 0800-LUC dual-luciferase reporter vector and introduced the resulting plasmid into Agrobacterium tumefaciens EHA105 (pSoup) to generate the reporter construct. For effectors, we employed the empty pEAQ vector and pEAQ-*CcWRKY31*. The reporter and effector constructs were co-transformed into tobacco (*Nicotiana benthamiana*) leaves at an effector-to-reporter ratio of 9:1. After 2–3 days, the LUC/REN luminescence ratio was determined. The experiment was performed with a minimum of six independent biological replicates [34].

### 4.12. Statistical Analysis

All experiments were conducted with three independent biological replicates under a randomized design. All statistical evaluations were conducted using SPSS version 25.0. The significance of differences between two groups was determined by a two-tailed Student’s *t*-test, whereas for comparisons across three or more groups, one-way analysis of variance (ANOVA) followed by Duncan’s multiple-range test was employed. Results with *p* < 0.05 were deemed statistically significant, and those with *p* < 0.01 were considered highly significant. Statistical analyses and graphical presentation were carried out using GraphPad Prism 10.0.

## Data Availability

The original contributions presented in this study are included in the article/Supplementary Materials. Further inquiries can be directed to the corresponding author.

## Supplementary Materials

The following supporting information can be downloaded at: https://www.mdpi.com/article/10.3390/ijms27198798/s1.
