# Supplementary material for: CcWRKY31 Regulates Postharvest Mandarin Fruit Coloration in a Temperature-Dependent Manner
Source: Int J Mol Sci. 2026 Oct 1;27(19):8798. doi: 10.3390/ijms27198798 (PMC13665423; doi:10.3390/ijms27198798)
Supplement: Supplementary file 1 [file ijms-27-08798-s001.zip › ijms-4551200-supplementary.pdf]

Supplementary

Table S1. Summary of primers used in this study.

Assay	Primer sequence
Subcellular localization	*CcWRKY31-pCAMBIA2300GFP-For: caatttactattctagggtaccATGGACAAAGGATGGGGT*<br>*CcWRKY31-pCAMBIA2300GFP-Rev: gtatgggtatctagactgcagTTGGTGTTGCCTGTTGTT*
Y2H	*CcWRKY31-pGBKT7-For: aggccgaattcccggggatccATGGACAAAGGATGGGGT*<br>*CcWRKY31-pGBKT7-Rev: ctagttatgcggccgctgcagTTGGTGTTGCCTGTTGTT*
RT-qPCR	*CcWRKY31-For: GGAATCTGAAACACAGGGAT*<br>*CcWRKY31-Rev: GTGCAACGATAATAAGCTCG*<br>*CcActin-For: TGGATTCTGGTGATGGTGTG*<br>*CcActin-Rev: GTTCGGCTGTGGTGGTAAAC*<br>*CcPSY-For: GATTCCTCAGCTTCTGCTC*<br>*CcPSY-Rev GTTTTCCTCTCGAACTGAATCG*<br>*CcCRTZ-For: GCTTCTTTAATGGCAAAAACCG*<br>*CcCRTZ-Rev: GGATCTCTTTCTCGCCAATTTC*<br>*CcLCYE-For: ACAAACAATTATGGTGTGTGGG*<br>*CcLCYE-Rev: TGTCGACTAACTCGTCCATAAG*<br>*CcCCS-For: CAACTCTTCTTAGCCCGTTTTC*<br>*CcCCS-Rev: CCAGGGGAGATCAAAGTCTAAG*<br>*CcZEP-For: CTAAAGAGCTATGAGAGAGCTAGGAGACT*<br>*CcZEP-Rev: CACTGCGGCCGATCTTG*<br>*CcNCED5-For: AGAGCGTTTTATCCGAAATTCG*<br>*CcNCED5-Rev: CTCGCCGCCATATTTATTATCG*<br>*CcNCED1-For: AACCCGTCTGCCAGAACCTT*<br>*CcNCED1-Rev: GTTGGCTCCGTTTCTGACGTA*<br>*CcCHLM-For: AAAGCGCCCATGAAAATCTATC*<br>*CcCHLM-Rev: AGTGATATTTTGTGTTTCCCGC*<br>*CcHMOX-For: GCTCTTTCTTCAAACTTCCGAG*<br>*CcHMOX-Rev: TTCTCTAGCCTGCTCCTTAGTA*<br>*CcCPOX-For: CATTTACCGATCAGCATAAGGC*<br>*CcCPOX-Rev: ACTCTCCAGATGATACAAAGGC*<br>*CcHEML-For: ATATCGATGGCAACGAGTACAT*<br>*CcHEML-Rev: CAGCTGAGATTACCATTTCTGC*<br>*CcNYC1-For: GGCACGGTTTTCCTTTACAGATG*<br>*CcNYC1-Rev: TGTTGTAGTTCTGACGCTTTCTG*<br>*CcRCCR-For: CAAACGTTGCAGGTCTTCGG*<br>*CcRCCR-Rev: CAAACGTTGCAGGTCTTCGG*
EMSA	*CcWRKY31-pGEX-For: ggttccgcgtggatccATGGACAAAGGATGGGGT*<br>*CcWRKY31-pGEX-Rev: agtcacgatgcggccgcTTGGTGTTGCCTGTTGTT*<br>*CcCRTZ-Probe-For: CCTTCAATGTTTTGTCAAATTGCATGAATGTACAGTACGG*<br>*CcCRTZ-Mutant-For: CCTTCAATGTTTTTTTTTATTTTTTGAATGTACAGTACGG*<br>*CcCRTZ-Probe-Rev: CCGTACTGTACATTCATGCAATTTGACAAAACATTGAAGG*<br>*CcCRTZ-Mutant-Rev: CCGTACTGTACATTCAAAAAATAAAAAAAAACATTGAAGG*<br>*CcLCYE-Probe-For: AATTGCAATCATGACCATGACCATGACCGTGTCTGCATC*<br>*CcLCYE-Mutant-For: AATTGCAATCATTTTTATTTTTATTTTTGTGTCTGCATC*<br>*CcLCYE-Probe-Rev: GATGCAGACACGGTCATGGTCATGGTCATGATTGCAATT*<br>*CcLCYE-Mutant-Rev: GATGCAGACACAAAAATAAAAATAAAAATGATTGCAATT*<br>*CcCCS-Probe-For: AAATTAGGGTCAGGTCAAATTGCTATTTCTAGTAGTGACTGTTGTAT*

	CcCCS-Mutant-For: AAATTAGTTTTTTTTTTTATTGCTATTTCTAGTAGTGACTGTTGTAT
	CcCCS-Probe-Rev: ATACAACAGTCACTACTAGAAATAGCAATTTGACCTGACCCTAATTT
	CcCCS-Mutant-Rev: ATACAACAGTCACTACTAGAAATAGCAATAAAAAAAAAAACTAATTT
	CcZEP-Probe-For: CTTTTTTTATCCCCACATATTTGTCAATACCAATTTACACAAGCT
	CcZEP-Mutant-For: CTTTTTTTATCCCCACATATTTTTTTTTTACCAATTTACACAAGCT
	CcZEP-Probe-Rev: AGCTTGTGTAAATTGGTATTGACAAATATGTGGGGATAAAAAAAG
	CcZEP-Mutant-Rev: AGCTTGTGTAAATTGGTAAAAAAAAAATATGTGGGGATAAAAAAAG
	CcNCED5-Probe-For: CTTTCTGTGGGCATGACTGTGTCTCTGACTCTAACTCTCACTC
	CcNCED5-Mutant-For: CTTTCTGTGGGCATTTTTGTGTCTCTTTTTCTAACTCTCACTC
	CcNCED5-Probe-Rev: GAGTGAGAGTTAGAGTCAGAGACACAGTCATGCCCACAGAAAG
	CcNCED5-Mutant-Rev: GAGTGAGAGTTAGAAAAAGAGACACAAAAATGCCCACAGAAAG
	CcNYC1-Probe-For: ATCAATATGTCAAGGAAATAAATGTCATTGACTAAGCCCAAA
	CcNYC1-Mutant-For: ATCAATATTTTTTGGAAATAAATTTTTTTTTTTAAGCCCAAA
	CcNYC1-Probe-Rev: TTTGGGCTTAGTCAATGACATTTATTTCCTTGACATATTGAT
	CcNYC1-Mutant-Rev: TTTGGGCTTAAAAAAAAAAATTTATTTCCAAAAAATATTGAT
	CcRCCR-Probe-For: TGTTTTTTTATTGAGTTGTCAATTGACATCTAATGATCC
	CcRCCR-Mutant-For: TGTTTTTTTATTGAGTTTTTTTTTTTTATCTAATGATCC
	CcRCCR-Probe-Rev: GGATCATTAGATGTCAATTGACAACTCAATAAAAAAACA
	CcRCCR-Mutant-Rev: GGATCATTAGATAAAAAAAAAAAACTCAATAAAAAAACA
DLR	*CcWRKY31-pEAQ-For: caaattcgcgaccggtATGGACAAAGGATGGGGT*
	CcWRKY31-pEAQ-Rev: agttaaaggcctcgagTTGGTGTTGCCTGTTGTT
	CcWRKY31-pBD-For: tcgccgaccggtaggcctATGGACAAAGGATGGGGT
	CcWRKY31-pBD-Rev: aaccagagttaaaggcctTTGGTGTTGCCTGTTGTT
	CcCRTZ-pro-0800-For: cactatagggcgaattggGTACATAATATCTAAAGTTGACC
	CcCRTZ-pro-0800-Rev: ttggcgtcttccatggTAAGTGTACAATTTTAATTGGAC
	CcLCYE-pro-0800-For: cactatagggcgaattggTCCAGATCATCCAAATGGCCGT
	CcLCYE-pro-0800-Rev: ttggcgtcttccatggACGACGCACCGTTCAACAAA
	CcCCS-pro-0800-For: cactatagggcgaattggGTCGGGTTGAACTTGGTCAA
	CcCCS-pro-0800-Rev: ttggcgtcttccatggATCCGCACTAAAGATATCAGCTAC
	CcZEP-pro-0800-For: cactatagggcgaattggTTAGCTAACTTTGATGCTAAT
	CcZEP-pro-0800-Rev: ttggcgtcttccatggTGATAATAGTAAAGCCGGTA
	CcNCED5-pro-0800-For: cactatagggcgaattggTTTCATCCTTCTTTGTCTGGT
	CcNCED5-pro-0800-Rev: ttggcgtcttccatggAGGGAGAAAACAAAATAAAGGG
	CcNYC1-pro-0800-For: cactatagggcgaattggTGGTCCACTCATGTTATAATACTG
	CcNYC1-pro-0800-Rev: ttggcgtcttccatggAAGCAGTAATAGTGCAAGGTTT
	CcRCCR-pro-0800-For: cactatagggcgaattggCCTATGGACTACACTATAAA
	CcRCCR-pro-0800-Rev: ttggcgtcttccatggCAATCGTGATAGTTAATGT

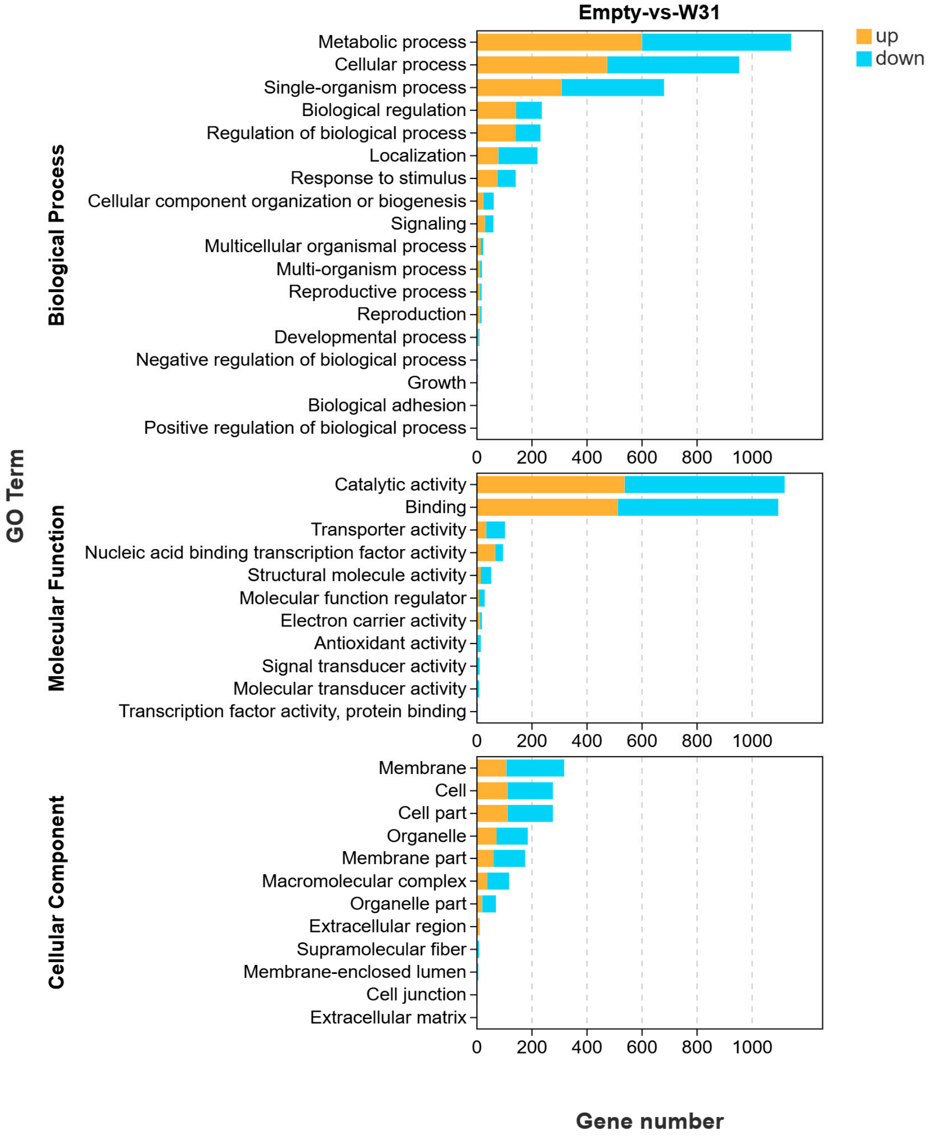

Figure S1. GO enrichment analysis.

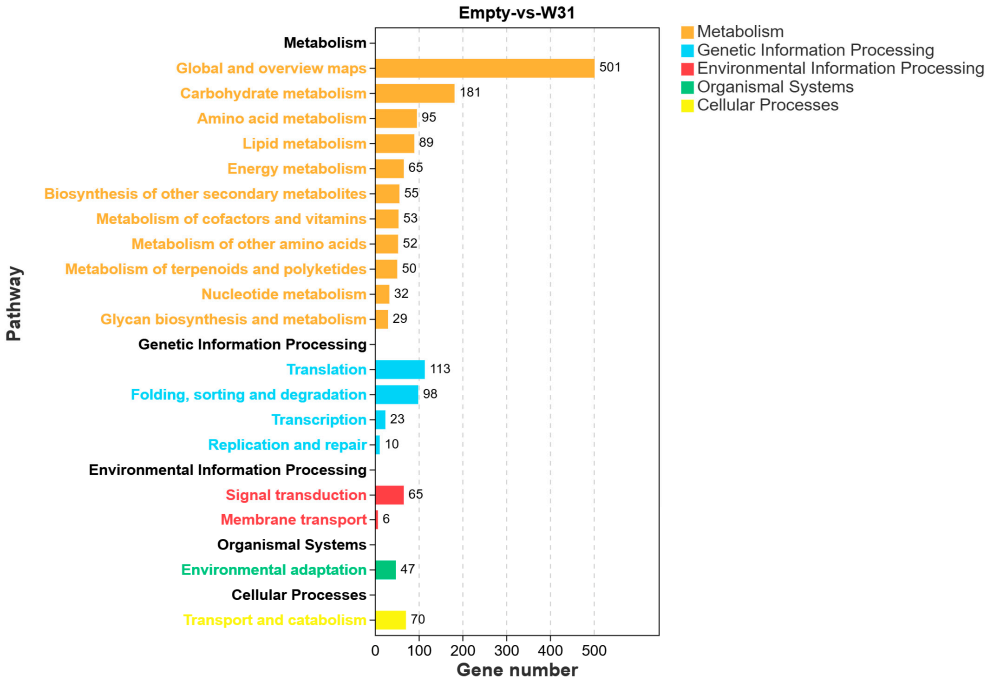

Figure S2. KEGG enrichment analysis.
